# Risk Factors for Community and Intrahousehold Transmission of SARS-CoV-2: Modeling in a Nationwide French Population-Based Cohort Study, the EpiCoV Study

**DOI:** 10.1093/aje/kwad174

**Published:** 2023-08-18

**Authors:** Sophie Novelli, Lulla Opatowski, Carmelite Manto, Delphine Rahib, Xavier de Lamballerie, Josiane Warszawski, Laurence Meyer, on behalf of the EpiCoV Study Group

**Keywords:** coronavirus disease 2019, COVID-19, disease transmission, households, population-based surveys, SARS-CoV-2, seroprevalence, severe acute respiratory syndrome coronavirus 2

## Abstract

We assessed the risk of acquiring severe acute respiratory syndrome coronavirus 2 (SARS-CoV-2) from household and community exposure according to age, family ties, and socioeconomic and living conditions using serological data from a nationwide French population-based cohort study, the Epidémiologie et Conditions de Vie (EpiCoV) Study. A history of SARS-CoV-2 infection was defined by a positive anti-SARS-CoV-2 enzyme-linked immunosorbent assay immunoglobulin G result in November–December 2020. We applied stochastic chain binomial models fitted to the final distribution of household infections to data from 17,983 individuals aged ≥6 years from 8,165 households. Models estimated the competing risks of being infected from community and household exposure. The age group 18–24 years had the highest risk of extrahousehold infection (8.9%, 95% credible interval (CrI): 7.5, 10.4), whereas the oldest (≥75 years) and youngest (6–10 years) age groups had the lowest risk, at 2.6% (95% CrI: 1.8, 3.5) and 3.4% (95% CrI: 1.9, 5.2), respectively. Extrahousehold infection was also associated with socioeconomic conditions. Within households, the probability of person-to-person transmission increased with age, from 10.6% (95% CrI: 5.0, 17.9) among children aged 6–10 years to 43.1% (95% CrI: 32.6, 53.2) among adults aged 65–74 years. Transmission was higher between partners (29.9%, 95% CrI: 25.6, 34.3) and from mother to child (29.1%, 95% CrI: 21.4, 37.3) than between individuals related by other family ties. In 2020 in France, the main factors identified for extrahousehold SARS-CoV-2 infection were age and socioeconomic conditions. Intrahousehold infection mainly depended on age and family ties.

## Abbreviations

aOR: adjusted odds ratio
COVID-19: coronavirus disease 2019
CrI: credible interval
ELISA: enzyme-linked immunosorbent assay
EpiCoV: Epidémiologie et Conditions de Vie
SARS-CoV-2: severe acute respiratory syndrome coronavirus 2

Monitoring of households, where individuals of different generations have close and repeated contacts, can help us understand the transmission of pathogens. Regarding coronavirus disease 2019 (COVID-19), household studies have provided accumulating evidence that susceptibility to severe acute respiratory syndrome coronavirus 2 (SARS-CoV-2) infection is lower for children under 8–10 years of age than for adults and higher among persons over ages 60–65 years than among younger/middle-aged adults (1, 2). However, evidence is still lacking regarding risk for newborns, young children, and adolescents, while these age groups are characterized by highly heterogeneous behavior and social contact patterns.

Based on a population-based serosurvey carried out in Geneva, Switzerland, during April–June 2020, Bi et al. (3) identified a reduced risk of intrahousehold infection among children aged 5–9 years and young people aged 10–19 years than among adults aged 20–49 years. However, that study was carried out at a time of particularly low prevalence among children across Europe (4–8) after a long period of school closure during which children had limited interactions outside the home, limiting the possibility of studying the different routes of infection and infectivity among children. During June–December 2020, data from a Canadian pediatric cohort highlighted higher infectivity among children than among adolescents (9). Importantly, despite growing evidence of social disparities in the risk of SARS-CoV-2 infection (10–14), socioeconomic factors were rarely evaluated at the household level.

The Epidémiologie et Conditions de Vie (EpiCoV) Study (included in the ORCHESTRA (Connecting European Cohorts to Increase Common and Effective Response to SARS-CoV-2 Pandemic) project) is based on a rich nationwide population-based cohort in France that combines serological testing and longitudinal follow-up and that aims to analyze the associations between living conditions and the dynamics of the COVID-19 epidemic in France. In November–December 2020, when schools were open and vaccination had not yet been implemented, we conducted a serological survey of households from the EpiCoV cohort. Serological assays are used to measure antibody responses in blood samples as the sign of a previous SARS-CoV-2 infection, while virological tests detect ongoing infection. While the latter tests may miss mild or asymptomatic infections (15), serological tests remain sensitive over a longer time period (16), and thus they consist of complementary tools with which to define infection history and study SARS-CoV-2 transmission. Here, we used mathematical modeling to assess, from the EpiCoV data, the associations of age, family ties, and living and socioeconomic conditions with the risk of SARS-CoV-2 acquisition from both household and community exposure.

## METHODS

### The EpiCoV cohort

EpiCoV is a nationwide population-based cohort study that combines serological testing and longitudinal follow-up. It aims to analyze both the impact of living conditions on the dynamics of the COVID-19 epidemic and the impact of the epidemic on health and living conditions in France. In May 2020, 371,000 individuals aged ≥15 years living in mainland France or 3 of the 5 French overseas territories were randomly selected from the Fidéli (Fichiers Démographiques sur les Logements et les Individus) administrative sampling frame. This database is considered to be quasi-exhaustive for the population living in France (17). The survey design was defined to ensure overrepresentation of the less densely populated departments and lower-income households, for which lower response rates were expected. Selected individuals were contacted to undergo a World Wide Web/telephone questionnaire. The survey design and multimodal data collection have been detailed elsewhere (11).

### Household study design

In November 2020, a 20% subsample of EpiCoV participants was randomly drawn to be part of a household study. Eligible participants were offered home capillary blood self-sampling for SARS-CoV-2 serology for all household members (i.e., any person aged >5 years living at that address). Only 1 household member per household, that is, the one initially sampled to be part of the EpiCoV cohort, completed the questionnaire. He/she was defined as the respondent member. Households in which the respondent was under age 17 years or living in a household with more than 9 members were not included in the household substudy.

### Epidemiologic context of the household survey

This household survey aimed to capture infections that had occurred from the start of the COVID-19 pandemic in France (February–March 2020) to November–December 2020. This period covered the first 2 waves of the pandemic, in which infection was mostly caused by the wild-type virus before the Alpha variant gradually became dominant after its introduction at the end of 2020 (18) and before the start of the vaccination campaign on December 27, 2020 (19). The epidemiologic evolution of the pandemic during the year 2020 in France has been described elsewhere (12) (see Web Appendix 1 and Web Figure 1, available at https://doi.org/10.1093/aje/kwad174).

### Laboratory analyses

Dried blood spots were collected on Whatman 903 Proteinsaver cards (Whatman GmbH, Dassel, Germany) sent to each participant who agreed to blood sampling and mailed to one of the 3 participating biobanks (in Bordeaux, Amiens, and Montpellier) to be punched using a Panthera-Puncher machine (PerkinElmer, Inc., Waltham, Massachusetts). Eluates were processed in the virology laboratory (Unité des Virus Emergents, Marseille) with a commercial enzyme-linked immunosorbent assay (ELISA) kit (EUROIMMUN Medizinische Labordiagnostika AG, Lübeck, Germany) for the detection of anti–SARS-CoV-2 antibodies (immunoglobulin G) against the S1 domain of the viral spike protein (ELISA-S), according to the manufacturer’s instructions.

### Outcome

SARS-CoV-2 seropositivity was defined as an ELISA-S immunoglobulin G ratio greater than or equal to 1.1, according to the ratio threshold supplied by the manufacturer, and was considered the main outcome.

### Exposures

Collected data included the number, age, and sex of all individuals living in the household and the decile income of the household per capita. We also considered the administrative region, the population density in the municipality of residence, whether the household was overcrowded (defined as housing with less than 18 m^2^ per inhabitant), and whether the neighborhood was defined as socially deprived according to national definitions for prioritizing targeted socioeconomic interventions.

### Ethics

The survey was approved by the French data protection authority (Commission Nationale de l’Informatique et des Libertés) and the local ethics committee (Comité de Protection des Personnes Sud Mediteranée). The survey was also reviewed by the Comité du Label de la Statistique Publique. The serological results were sent to the participants by mail with information on how to interpret the individual test results.

### Statistical analysis

We analyzed all households for which serostatus data were available for all members. Because serostatus information on young children aged ≤5 years was not available, households with children aged ≤5 years were removed. We applied stochastic chain binomial transmission models to the final distribution of infections in households (3, 20). This type of model is suited to the analysis of results of a seroprevalence survey when only the final distribution (i.e., the serostatus of each household member at the time of the survey) is available, without any information about the chronology of infection between individuals. Data augmented with all possible sequences of virus introduction into each household and subsequent transmission events within the household are therefore considered. In other words, for each seropositive household member, the possibility that he or she was infected either by the community or by another infected household member was considered in different sequences, and the probability of each sequence could be determined (see Web Appendix 2 and Web Figure 2). This allowed us to estimate 1) the probability that a susceptible individual *i* had been infected through extrahousehold exposure from the beginning of the pandemic to the time of the serosurvey and 2) the probability that a susceptible individual *i* contracted infection from a single infectious household member *j* (note that this was not the overall probability of being infected within the household but a probability of person-to-person transmission of SARS-CoV-2).

For simplicity purposes, we considered serological status to be a perfect marker for having been infected and neglected the occurrence of false-positive or false-negative results. We also neglected the possibility that individuals could have been infected several times. These 2 points will be addressed below in the Discussion section.

We investigated factors associated with the risk of extra- and intrahousehold SARS-CoV-2 acquisition. They included the characteristics of the susceptible individual 𝑖 and the potential infector 𝑗, as well as the living and socioeconomic conditions of the household. The variables considered for modeling the probability of extrahousehold acquisition were age and sex of the susceptible household member, family income, population density in the municipality of residence, and living in a socially deprived neighborhood. We also considered immigration status, information on this variable being collected for the respondent. We accounted for spatial heterogeneity in the extrahousehold probability, adjusting for the administrative region. For the probability that an infectious household member *j* infected a susceptible household member *i*, we considered the following covariates: age and sex of the susceptible individual *i*, age and sex of the potential infector *j*, family ties between *i* and *j* (i.e., whether *j* was *i*’s partner, parent, child, sibling, grandparent, grandchild, or other), household size, accommodation type, and number of rooms. We also tested whether demographic and socioeconomic factors (immigration status, family income, population density in the municipality of residence, and living in a socially deprived neighborhood) and administrative region modulated the risk of intrahousehold transmission.

We built a series of models including various combinations of these variables. Model parameters were estimated by Bayesian inference via Markov chain Monte Carlo sampling*.* Model fits were compared using the widely applicable information criterion (21). Given the very low percentage of missing data for the considered variables (<4%), we conducted complete-case analysis.

We adapted the software code written by Bi et al. (3) and available in open access to the EpiCoV data. Models were implemented in the Stan probabilistic programming language using the “rstan” package (version 2.21.2) in R (R Foundation for Statistical Computing, Vienna, Austria). We used weakly informative priors on all parameters to be normally distributed on the logit scale, with a mean of 0 and a standard error of 1.5. We ran 4 chains of 1,500 iterations each with 500 warm-up iterations. We assessed convergence visually and using the Gelman-Rubin convergence statistic (^*R*). We report estimates as the median values of the posterior samples with 95% credible intervals (CrIs), that is, the 2.5th and 97.5th percentiles of their distributions. See Web Appendix 2, Web Figures 3 and 4, and Web Table 1 for adequacy checking and model validation.

## RESULTS

### Study population

**Figure 1:**
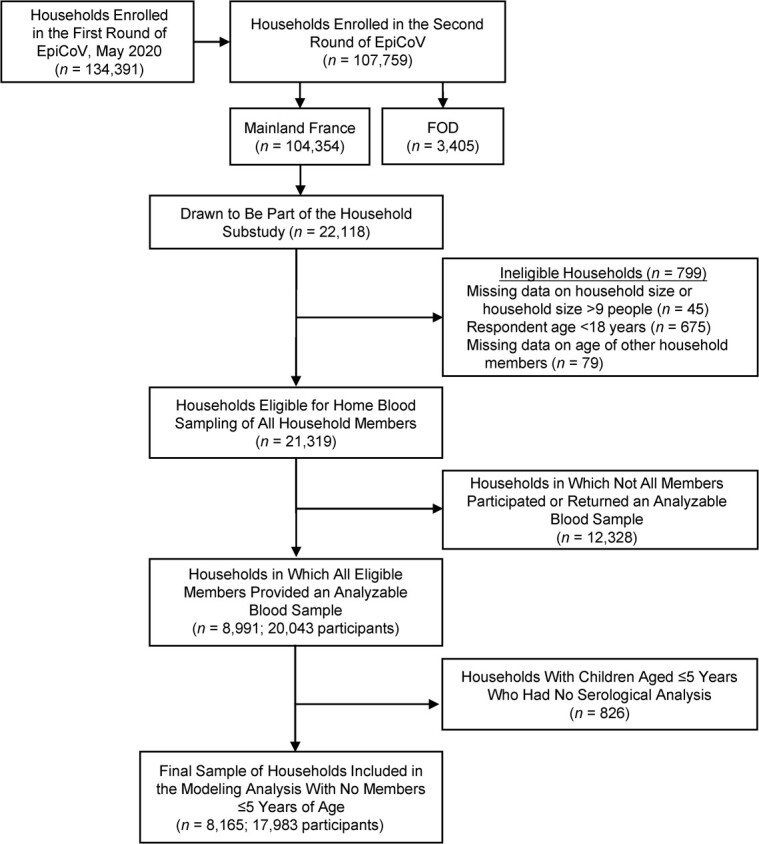
Enrollment of participants in the EpiCoV Study and its household substudy, 2020. EpiCoV, Epidémiologie et Conditions de Vie; FOD, French overseas departments.

Among the 22,118 households in mainland France that were drawn from the EpiCoV cohort to be part of the household substudy in November 2020, we analyzed 17,983 individuals belonging to 8,165 households for which the serostatus of all members was available and all members were over age 5 years (Figure 1). The median date of blood sampling was November 27, 2020 (interquartile range, November 23–December 6). The median age of the participants was 52 (interquartile range, 28–64) years, and the male:female sex ratio was 0.93. The characteristics of the households are shown in Table 1. Most of the households were 1-person (25.9%) or 2-person (47.4%) households, which differed from the general population (36.9% and 32.6% were 1- and 2-person households in 2019, respectively (22)). The main family structures were couples without (43.2%) and with (23.8%) children. Young people (ages 6–24 years) and middle-aged adults (ages 35–54 years) mainly lived in households with more than 2 members, whereas adults aged 25–34 and ≥55 years mainly lived in 2-person households (see Web Table 2). The proportion of 1-person households was highest among persons aged 25–34 years (21.1%) and ≥75 years (21.0%).

**Table 1:** Characteristics of Households in the Household Substudy of the EpiCoV Study, 2020

**Characteristic**	**Missing Data**		**Household Data (*n* = 8,165)**	
	**%**	**No.**	**%**	**No.**
Household size, no. of persons	0	0		
1			25.9	2,116
2			47.4	3,870
3			11.5	941
4			11.5	937
≥5			3.7	301
Family structure	0	0		
Living alone			25.9	2,116
Couple without children			43.2	3,524
Single-parent family			4.7	385
Couple with 1 or more children			23.8	1,941
3-generation family			0.2	20
Other household structure			2.2	179
Family income per capita (deciles)	1.9	153		
1 (lowest)			5.7	458
2–3			9.9	794
4–5			13.6	1,093
6–7			20.8	1,663
8–9			31.5	2,523
10 (highest)			18.5	1,481
Population density in municipality of residence^a^	0	0		
Low			37.2	3,038
Medium			28.9	2,358
High			33.9	2,769
Living in a socially deprived neighborhood	0	0	2.2	176
Accommodation type	<0.1	2		
Apartment with no balcony, terrace, or community garden			9.4	764
Apartment with a balcony or terrace			18.9	1,545
Apartment with a community garden			2.3	189
House with a yard or garden			0.9	70
House with no yard or garden			67.9	5,546
Other			0.6	49
Housing overcrowding	6.5	528		
Living alone			21.8	1,665
Housing not particularly crowded			74.5	5,688
Crowded housing			3.7	284
Region	0	0		
Occitanie			10.7	870
Nouvelle-Aquitaine			9.3	761
Hauts-de-France			9.0	731
Île-de-France			16.3	1,327
Bretagne			5.4	444
Auvergne-Rhône-Alpes			12.8	1,045
Provence-Alpes-Côte-d’Azur			6.4	525
Grand Est			10.1	828
Normandie			4.5	369
Centre-Val de Loire			4.2	343
Bourgogne-Franche-Comté			4.7	386
Corse			0.4	31
Pays de la Loire			6.2	505
Immigration status of respondent^b^	3.1	254		
Majority population			87.8	6,946
Immigrant from Europe				
First-generation			2.9	227
Second-generation			5.2	409
Immigrant from outside Europe				
First-generation			1.8	145
Second-generation			2.3	184

Abbreviation: EpiCoV, Epidémiologie et Conditions de Vie.

^a^ Population density in the municipality of residence was defined on the basis of a grid cell classification of urbanization (42). High density: a municipality where more than 50% of the population lives in an urban center (a cluster of contiguous grid cells of 1 km² with a population density ≥1,500 inhabitants/km² and ≥50,000 inhabitants overall). Medium density: a municipality where more than 50% of people live in an urban center or urban cluster (a cluster of contiguous grid cells of 1 km² with a population density ≥300 inhabitants/km² and ≥5,000 inhabitants overall). Low density: any other area.

^b^ The respondent participant was the household member who was sampled to be part of the EpiCoV Study and answered the questionnaire.

### Seroprevalence and household final attack rates

**Figure 2:**
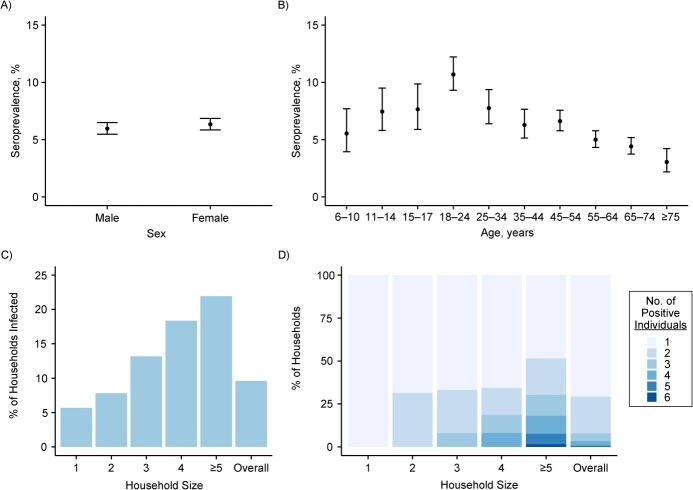
Characteristics of participants in the household substudy of the EpiCoV Study, 2020. A) SARS-CoV-2 seroprevalence (bars show 95% confidence intervals (CIs)) according to sex. The analysis included 8,674 males and 9,293 females. B) SARS-CoV-2 seroprevalence (bars, 95% CIs) according to age. Numbers of individuals by age group: ages 6–10 years, *n* = 632; ages 11–14 years, *n* = 832; ages 15–17 years, *n* = 745; ages 18–24 years, *n* = 1,797; ages 25–34 years, *n* = 1,304; ages 35–44 years, *n* = 1,513; ages 45–54 years, *n* = 3,100; ages 55–64 years, *n* = 3,601; ages 65–74 years, *n* = 3,244; ages ≥75 years, *n* = 1,215. C) Proportion of infected households (i.e., households with at least 1 positive case) according to household size. The overall number of households was 8,165. Numbers of households by household size: 1 person, *n* = 2,116; 2 people, *n* = 3,870; 3 people, *n* = 941; 4 people, *n* = 937; ≥5 people, *n* = 301. D) Among infected households (i.e., households with at least 1 positive case), total number of positive individuals in the household according to the size of the household. For example, among 3-member infected households, 67% had only 1 positive household member, 25% had 2 positive members, and 8% had 3 positive members. The overall number of infected households was 784. Numbers of infected households by household size: 1 person, *n* = 66; 2 people, *n* = 120; 3 people, *n* = 302; 4 people, *n* = 124; ≥5 people, *n* = 172. EpiCoV, Epidémiologie et Conditions de Vie; SARS-CoV-2, severe acute respiratory syndrome coronavirus 2.

At the individual level, overall seroprevalence did not differ by sex but varied with age, increasing from 5.5% among children aged 6–10 years to 7.5% among children aged 11–14 years and 7.7% among children aged 15–17 years, before peaking at 10.7% among young adults aged 18–24 years and then decreasing to 3.0% among adults aged ≥75 years (Figures 2A and2B).

The proportion of households with at least 1 seropositive member was 9.6% overall and increased from 5.7% for 1-person households to 22.0% for households with ≥5 members (Figure 2C, Web Table 3). Most infected households counted only 1 or 2 positive individuals, even in households of 4 or more people (Figure 2D). Among households with at least 1 positive individual, the proportion of positive members was 52% overall, and it decreased with household size, from 66% in 2-person households to 47% in 3-person households to 40% in 4- and ≥5-person households.

### Factors associated with SARS-CoV-2 acquisition

#### Infection from extrahousehold exposure.

**Figure 3:**
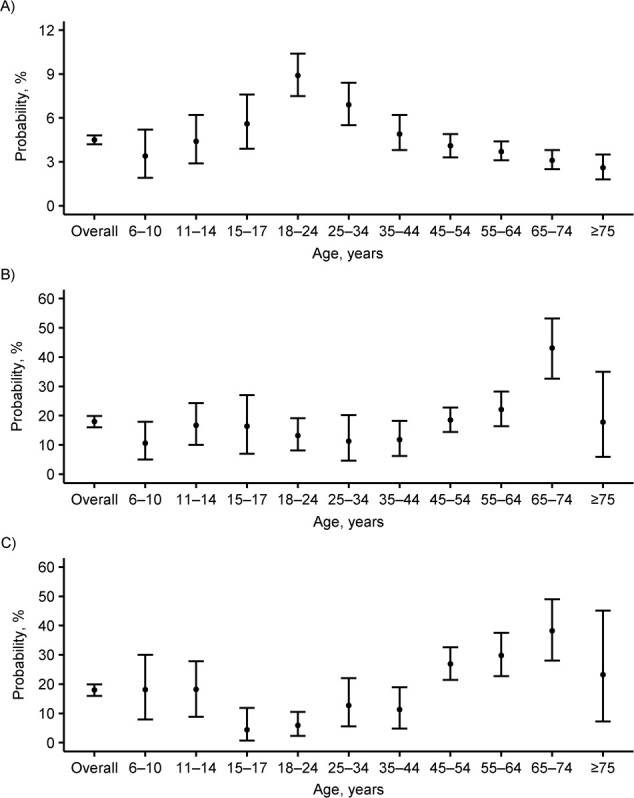
Estimated probabilities (median values with 95% credible intervals (bars)) of extra- and intrahousehold SARS-CoV-2 infection, by age, in the household substudy of the EpiCoV Study, 2020. A) Estimated probability of infection from extrahousehold exposure according to the age of the susceptible individual. B) Estimated probability of infection from 1 single infected household member according to the age of the susceptible. C) Estimated probability of infection from 1 single infected household member according to the age of the infector. EpiCoV, Epidémiologie et Conditions de Vie; SARS-CoV-2, severe acute respiratory syndrome coronavirus 2.

We estimated the overall cumulative probability of infection due to extrahousehold exposure from the start of the pandemic in France through the time of the survey to be a median of 4.5% (95% CrI: 4.2, 4.9). This probability varied with age: Young adults aged 18–24 years had the highest probability of extrahousehold infection and adults aged ≥75 years had the lowest (Figure 3A). In multivariate analysis, the probability of being infected through extrahousehold exposure was adjusted for age of the susceptible individual, family income, population density in the municipality of residence, immigration status of the respondent, and administrative region of residence. The association with age remained similar: Young people (ages 18–24 years) and younger adults (ages 25–34 and 35–44 years) had a higher risk of extrahousehold SARS-CoV-2 acquisition than did middle-aged adults (ages 55–64 years) (Table 2). The probability of extrahousehold infection increased with family income (highest for the 2 highest deciles as compared with the central deciles) and population density in the municipality of residence. Individuals in households in which the respondent participant was a first- or second-generation immigrant from outside Europe had a higher risk of extrahousehold SARS-CoV-2 acquisition in univariate analysis. This association was tempered after adjustment for socioeconomic factors. Finally, as expected, the regions with the highest SARS-CoV-2 incidence at the time of the survey were those with the highest probability of extrahousehold infection.

**Table 2:** Factors Associated With Risks of Extrahousehold and Intrahousehold SARS-CoV-2 Infection in the EpiCoV Study, 2020

	**Infection From Extrahousehold Exposure**				**Infection From 1 Single Infected Household Member**			
	**Univariate Analysis**		**Multivariate Analysis**		**Univariate Analysis**		**Multivariate Analysis**	
**Variable**	**OR**	**95% CrI**	**OR**	**95% CrI**	**OR**	**95% CrI**	**OR**	**95% CrI**
Age of the susceptible, years								
6–10	0.9	0.5, 1.5	1.0	0.5, 1.6	0.4	0.2, 0.8	0.4	0.1, 1.2
11–14	1.2	0.8, 1.8	1.1	0.6, 1.6	0.7	0.4, 1.3	0.9	0.3, 2.1
15–17	1.5	1.0, 2.2	1.4	0.9, 2.1	0.7	0.3, 1.4	1.1	0.4, 2.8
18–24	2.5	2.0, 3.3	2.4	1.8, 3.1	0.5	0.3, 0.9	0.9	0.4, 2.0
25–34	1.9	1.4, 2.5	1.6	1.2, 2.2	0.5	0.2, 1.0	0.5	0.2, 1.1
35–44	1.3	1.0, 1.8	1.4	1.0, 1.9	0.5	0.2, 0.9	0.5	0.2, 1.1
45–54	1.1	0.8, 1.4	1.2	0.9, 1.6	0.8	0.5, 1.3	1.0	0.6, 1.9
55–64	1.0	Referent	1.0	Referent	1.0	Referent	1.0	Referent
65–74	0.8	0.6, 1.1	0.9	0.7, 1.2	2.7	1.5, 4.7	1.9	1.0, 3.5
≥75	0.7	0.5, 1.0	0.7	0.5, 1.0	0.8	0.2, 2.0	0.7	0.2, 2.1
Sex of the susceptible								
Female	1.0	Referent			1.0	Referent		
Male	0.9	0.8, 1.1			0.7	0.5, 1.0		
Age of the infector, years								
6–10					0.5	0.2, 1.1		
11–14					0.5	0.2, 1.1		
15–17					0.1	0.0, 0.3		
18–24					0.2	0.1, 0.3		
25–34					0.3	0.1, 0.7		
35–44					0.3	0.1, 0.6		
45–54					0.9	0.5, 1.4		
55–64					1.0	Referent		
65–74					1.5	0.8, 2.6		
≥75					0.7	0.2, 2.0		
Sex of the infector								
Female					1.0	Referent		
Male					0.8	0.5, 1.3		
Family ties (type of transmission)								
Between partners					1.0	Referent	1.0	Referent
From mother to child					1.0	0.6, 1.5	1.2	0.5, 2.8
From father to child					0.4	0.1, 0.7	0.3	0.1, 1.0
From child aged <12 years to parent					0.3	0.1, 0.8	0.6	0.1, 2.2
From child aged ≥12 years to parent					0.1	0.0, 0.2	0.3	0.1, 0.6
From grandparent to grandchild					0.5	0.0, 4.8	0.6	0.0, 6.7
From grandchild to grandparent					0.4	0.0, 2.6	0.5	0.0, 4.5
From sibling aged <12 years					0.4	0.1, 1.1	0.8	0.1, 3.0
From sibling aged ≥12 years					0.3	0.2, 0.6	0.4	0.1, 1.0
Between individuals with other family ties					0.4	0.1, 1.3	0.5	0.1, 1.5
Between individuals with no family ties					0.1	0.0, 0.7	0.2	0.0, 1.0
Immigration status of respondent^a^								
Majority population	1.0	Referent	1.0	Referent	1.0	Referent		
Immigrant from Europe								
First-generation	0.8	0.5, 1.2	0.8	0.5, 1.2	1.0	0.4, 2.1		
Second-generation	1.6	1.0, 2.4	1.3	0.8, 2.0	0.6	0.2, 1.2		
Immigrant from outside Europe								
First-generation	1.0	0.7, 1.4	1.0	0.7, 1.4	1.3	0.7, 2.3		
Second-generation	1.6	1.0, 2.3	1.4	0.9, 2.0	1.3	0.7, 2.4		
Family income per capita (deciles)								
1 (lowest)	1.2	0.8, 1.8	1.0	0.7, 1.5	0.5	0.2, 1.2		
2–3	1.0	0.7, 1.4	0.9	0.7, 1.3	1.3	0.7, 2.3		
4–5	1.0	Referent	1.0	Referent	1.0	Referent		
6–7	1.2	0.9, 1.5	1.2	0.9, 1.6	1.1	0.7, 1.9		
8–9	1.3	1.1, 1.7	1.3	1.0, 1.7	1.1	0.7, 1.7		
10 (highest)	1.7	1.3, 2.2	1.6	1.2, 2.1	1.4	0.9, 2.3		
Population density in municipality of residence^b^								
Low	1.0	Referent	1.0	Referent	1.0	Referent		
Medium	1.2	1.0, 1.4	1.1	0.9, 1.3	1.0	0.7, 1.4		
High	1.6	1.4, 1.9	1.2	1.0, 1.4	1.0	0.7, 1.4		
Household size, no. of persons								
2					1.0	Referent	1.0	Referent
3					0.5	0.3, 0.7	0.8	0.4, 1.4
4					0.4	0.3, 0.6	0.8	0.4, 1.3
≥5					0.4	0.3, 0.6	0.9	0.5, 1.7
Accommodation type								
Apartment with no balcony, terrace, or community garden					1.0	Referent		
Apartment with a balcony or terrace					1.5	0.4, 4.0		
Apartment with a community garden					1.3	0.9, 1.8		
House with a yard or garden					0.8	0.1, 2.5		
House with no yard or garden					1.1	0.5, 2.1		
Other					0.9	0.2, 3.1		
Housing overcrowding								
Housing not particularly crowded					1.0	Referent		
Crowded housing					1.2	0.8, 1.7		
Region								
Auvergne-Rhône-Alpes	1.9	1.3, 2.9	1.8	1.2, 2.7				
Bourgogne-Franche-Comté	1.1	0.7, 1.9	1.1	0.7, 1.9				
Bretagne	0.6	0.3, 1.0	0.5	0.3, 0.9				
Centre-Val de Loire	1.0	Referent	1.0	Referent				
Corse	0.2	0.0, 1.2	0.3	0.0, 1.6				
Grand Est	1.5	1.0, 2.3	1.5	1.0, 2.3				
Hauts-de-France	1.8	1.2, 2.7	1.8	1.2, 2.8				
Île-de-France	2.3	1.6, 3.5	1.8	1.2, 2.6				
Normandie	1.1	0.7, 1.9	1.1	0.6, 1.8				
Nouvelle-Aquitaine	0.8	0.5, 1.3	0.8	0.5, 1.3				
Occitanie	0.9	0.6, 1.4	0.9	0.6, 1.4				
Pays de la Loire	1.0	0.6, 1.6	1.0	0.6, 1.6				
Provence-Alpes-Cote d’Azur	0.8	0.5, 1.4	0.8	0.5, 1.3				

Abbreviations: CrI, credible interval; EpiCoV, Epidémiologie et Conditions de Vie; OR, odds ratio; SARS-CoV-2, severe acute respiratory syndrome coronavirus 2.

^a^ The respondent participant was the household member who was sampled to be part of the EpiCoV Study and answered the questionnaire.

^b^ Population density in the municipality of residence was defined on the basis of a grid cell classification of urbanization (42). High density: a municipality where more than 50% of the population lives in an urban center (a cluster of contiguous grid cells of 1 km² with a population density ≥1,500 inhabitants/km² and ≥50,000 inhabitants overall). Medium density: a municipality where more than 50% of people live in an urban center or urban cluster (a cluster of contiguous grid cells of 1 km² with a population density ≥300 inhabitants/km² and ≥5,000 inhabitants overall). Low density: any other area.

#### Infection from intrahousehold exposure.

**Figure 4:**
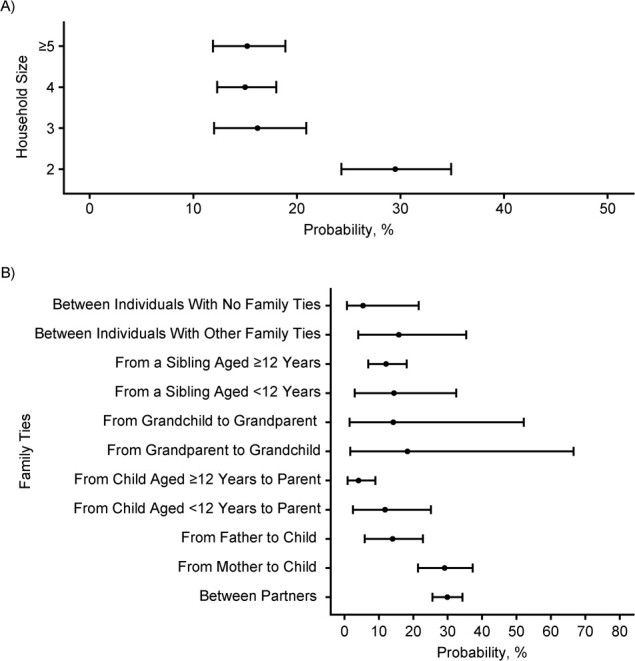
Estimated probability (median values with 95% credible intervals (bars)) of person-to-person SARS-CoV-2 transmission according to A) household size and B) family ties (type of transmission) in the household substudy of the EpiCoV Study, 2020. EpiCoV, Epidémiologie et Conditions de Vie; SARS-CoV-2, severe acute respiratory syndrome coronavirus 2.

The estimated median probability of person-to-person transmission between household members was 17.9% (95% CrI: 16.0, 19.9) overall. It varied with the age of the susceptible individual: Susceptibility was highest among adults aged 65–74 years and lowest among children aged 6–10 years (Figure 3B, Web Table 4). Models allowing for differential risk of transmission by the age of the infector showed the lowest infectivity for the age groups 15–17 and 18–24 years (Figure 3C, Web Table 5). The probability of person-to-person transmission between household members was 29.5% (95% CrI: 24.3, 34.9) in 2-person households and decreased to 15% in larger households (Figure 4A). These overall estimates concealed heterogeneous probabilities of transmission according to family ties. The probability of transmission was highest between partners (29.9%, 95% CrI: 25.6, 34.3), which was consistent with the estimate in 2-person households (Figure 4B). The probability of transmission was higher from mother to child (29.1%, 95% CrI: 21.4, 37.3) than from father to child (14.0%, 95% CrI: 5.9, 22.8). For transmission from child to parent, the probability was 11.8% (95% CrI: 2.5, 25.1) for children under 12 years of age and decreased to 4.1% (95% CrI: 0.9, 9.0) for children aged ≥12 years. The limited number of 3-generation families in the sample led to wide 95% CrIs when estimating the transmission risk between grandchildren and grandparents. Family income, population density in the municipality of residence, immigration status, and region were not associated with the risk of person-to-person transmission between household members (Web Tables 6–8).

In multivariate analysis, the probability of person-to-person transmission was adjusted for the age of the susceptible individual and his/her family link with the potential infector. Persons aged 65–74 years had twice the odds of being infected from a single infected household member than those aged 55–64 years (adjusted odds ratio (aOR) = 1.9, 95% CrI: 1.0, 3.5). Children aged 6–10 years appeared to have a lower risk of infection (aOR = 0.4, 95% CrI: 0.1, 1.2), but the 95% CrI was wide (Table 2). The risk of transmission from mother to child was similar to that between partners (aOR = 1.2, 95% CrI: 0.5, 2.8), whereas it was lower from father to child than from mother to child (aOR = 0.3, 95% CrI: 0.1, 1.0). The risk of transmission from child to parent decreased with increasing age of the child (aOR = 0.6 (95% CrI: 0.1, 2.2) for children aged <12 years and aOR = 0.3 (95% CrI: 0.1, 0.6) for children aged ≥12 years), using transmission between partners as the reference group. When adjusting for family ties, household size was no longer associated with the risk of person-to-person transmission.

Interestingly, variables pertaining to living conditions (i.e., type of accommodation and overcrowded housing) were not associated with the risk of person-to-person transmission in univariate analysis. The introduction of these 2 variables into the multivariate analysis did not change the odds ratio estimates for age or family ties.

### Proportion of intrahousehold infections

**Figure 5:**
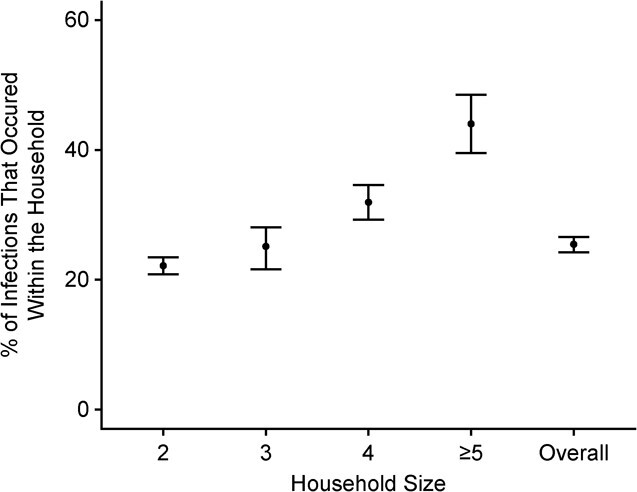
Estimated proportion of SARS-CoV-2 infections (median values with 95% credible intervals (bars)) that occurred within a household, according to household size, in the household substudy of the EpiCoV Study, 2020. EpiCoV, Epidémiologie et Conditions de Vie; SARS-CoV-2, severe acute respiratory syndrome coronavirus 2.

Using the posterior distribution of parameters from the final multivariate model, we simulated the predicted sources of infection for all individuals in the study (Web Appendix 1). We estimated that 25.5% (95% CrI: 25.1, 25.8) of all infections were caused by another household member. This proportion increased with household size, from 22.2% (95% CrI: 21.6, 22.7) for 2-person households to 44.0% (95% CrI: 42.5, 45.5) for households with ≥5 members (Figure 5).

## DISCUSSION

Using serological data from a nationwide population-based French cohort study, the EpiCoV Study, we estimated the risk of SARS-CoV-2 acquisition from the community and the person-to-person transmission risk within households in 2020. All participants over the age of 5 years were included, and we particularly investigated the associations of family composition, socioeconomic factors, and living conditions with intrahousehold SARS-CoV-2 transmission. Based on simulations, we found that household transmission represented one-quarter of SARS-CoV-2 infections. The main factors for extrahousehold infection were age and demographic and socioeconomic factors (i.e., family income, population density, and region), whereas intrahousehold infection mainly depended on the age of individuals and family ties between them.

Our results regarding an age pattern of SARS-CoV-2 transmission were consistent with previous household modeling studies (3, 23). Young adults had the highest probability of extrahousehold infection, probably reflecting a higher intensity of social interaction in this age group. The highest probability of being infected when exposed to an infectious individual at home was observed for the age group 65–74 years, which may be explained either by a higher susceptibility to infection or by greater time spent at home relative to the younger age groups, or by living with a partner of the same age who, if infected, was likely to be more infectious than younger people. Surprisingly, participants aged ≥75 years did not have a particularly higher risk of being infected by an infectious family member at home. This may reflect a potentially higher level of preventive measures in these populations. A higher level of waning antibodies in the oldest individuals may have also led to underestimation of the infection rate in this age group (24). Importantly, individuals living in nursing homes were not included in the EpiCoV Study.

Age-specific probabilities of person-to-person transmission result from a combination of biological effects (immune response and viral shedding) and behavioral factors (differences in social exposure and the frequency and intensity of contacts between age groups in the population). In models accounting for family ties, the probability of intrahousehold transmission was highest between partners and from mother to child. Lower values were obtained for transmission from a child to parents. This is consistent with the higher secondary infection rates for spouses than for other adult relationships, as reported previously (25, 26). We found a higher probability of transmission to parents from children aged ≤12 years than from older children, probably reflecting heterogeneity in contact patterns between individuals within families. Overall, our results suggest that in a context where schools were open, adults rather than children were more likely to be infected outside the household and to introduce the virus into the household. This is in line with previous in-depth investigations of transmission chains which indicated a limited role of children in SARS-CoV-2 transmission early in the pandemic (27–30). However, the relative burden of children was reported to increase during the subsequent COVID-19 waves caused by highly transmissible variants of concern (31), raising questions about changes in the susceptibility and infectivity of children with the emergence of variants of concern (32). When the wild-type virus predominated during 2020, household studies found lower household secondary attack rates in children than in adults, whereas in subsequent studies no significant difference was found between children and adults, supporting the hypothesis of increased susceptibility (and/or transmission) in children during the COVID-19 waves driven by the variants of concern (33). These changes may have resulted from a combination of factors associated with the evolution of SARS-CoV-2 epidemiology over time: 1) mass vaccination implemented at the beginning of 2021 targeting adults only (children were not eligible before June 2021 for those aged 12–17 years and December 2021 for those aged 5–11 years, and low vaccination coverage was achieved in children afterwards (34)); 2) the emergence of more transmissible variants; and 3) changes in the population mixing over time (35–37).

Family income and population density in the municipality of residence were associated with an increased probability of extrahousehold infection but not intrahousehold infection, which allows a better understanding of the association of these factors with the seroprevalence previously described in the EpiCoV cohort (11, 12). Surprisingly, we did not find associations between within-household person-to-person transmission and overcrowded housing or accommodation type, in opposition to some previous studies which reported associations between housing surface area and household secondary attack rates (38, 39). This difference may be due to the fact that relatively few dwellings were overcrowded in our sample. Isolation measures taken in households after a member became infected may also explain our results, but this information was not available.

The probability of person-to-person transmission was inversely related to household size. It was 30% in 2-person households, which corresponds to the estimated probability of transmission between partners. It decreased to 15%–16% in larger households, which is an average of all of the probabilities of transmission between household members, which ranged from 4% to 30% according to family ties. When adjusted for family ties, household size was no longer associated with the risk of person-to-person transmission.

Interestingly, from our simulations we estimated that household transmission represented one-quarter of SARS-CoV-2 infections in 2020. This value depends on the person-to-person probability of transmission within households, as well as the structure of the population: The larger the household size and the number of positive household members, the more likely are sequences involving 1 or more within-household infection events. In France, nearly 50% of households are 2-person households. Our study population mainly consisted of 1- and 2-person households (73%). The contribution of household transmission would be expected to be higher in settings where larger households are more frequent, as the estimated risk of household transmission increased from 22.2% in 2-person households to 44.0% in households with 5 or more members.

Among the main strengths of our study, in addition to its nationwide dimension, is that it covered a period of interest when schools were no longer closed in France, starting from autumn 2020, and children and young people were therefore more exposed to SARS-CoV-2 in the community. Furthermore, we accounted for competing risks between extrahousehold and intrahousehold exposure for SARS-CoV-2 acquisition and the possibility of tertiary transmission chains within households by using chain binomial models.

Some limitations need to be mentioned. First, despite the size of our study, one of the largest in Europe, its statistical power for the analysis of specific interactions (e.g., between grandparents and grandchildren) was limited. Second, antibody responses are generally sustained over the first 4 months following infection but may wane afterwards. Because the study took place in fall 2020, some infections that occurred early in the first wave of the pandemic may have been missed (24). Third, the possibility of reinfection was neglected here. Given that a single viral strain circulated at this time of the pandemic, this phenomenon was considered infrequent (24). Fourth, lack of availability of data on serological status among children aged ≤5 years impeded us from investigating the role of very young children in SARS-CoV-2 transmission. Finally, our results must be interpreted within the epidemiologic context of the study period. The pandemic has drastically changed since then, with both the emergence of variants of concern and the widespread implementation of vaccination and easing of containment measures, all of which could modify the association of age and other factors with transmission (40, 41).

In conclusion, our study brings new insights to the understanding of factors associated with the heterogeneity of intrahousehold SARS-CoV-2 transmission. It provides estimates of the extra- and intrahousehold risks of acquisition of SARS-CoV-2 over the first 2 waves of the COVID-19 pandemic in France in 2020. The probability of person-to-person transmission within households was estimated to be 18% overall, and it varied highly depending on the ages of the individuals and family ties. Our study illustrates the strength of the use of population-based serosurveys to assess the relative contributions of household and community transmission, which can be extended to the study of other respiratory viruses.

## Supplementary Material

Web_Material_kwad174
