# Supplementary material for: Risk Factors for Community and Intrahousehold Transmission of SARS-CoV-2: Modeling in a Nationwide French Population-Based Cohort Study, the EpiCoV Study
Source: Am J Epidemiol. 2023 Aug 18;193(1):134–48. doi: 10.1093/aje/kwad174 (PMC10773479; doi:10.1093/aje/kwad174)
Supplement: Web_Material_kwad174 [file web_material_kwad174.pdf]

WEB MATERIAL

Risk Factors for Community and Intrahousehold Transmission of SARS-CoV-2: Modeling in a Nationwide French Population-Based Cohort Study, the EpiCoV Study

Sophie Novelli, Lulla Opatowski, Carmelite Manto, Delphine Rahib, Xavier de Lamballerie, Josiane Warszawski, and Laurence Meyer on behalf of the EpiCoV Study Group

Table of Contents

Web Appendix 1. The COVID-19 pandemic in France in 2020 2

Web Appendix 2. Technical summary 3

Web Figure 1. A) Number of reported cases in France in 2020 (source WHO Coronavirus (COVID-19) Dashboard (5) and B) recruitment period 9

Web Figure 2. All possible sequences of viral introduction to each household and subsequent transmission events within the household for a household with three members with two positive individuals. 10

Web Figure 3. Model validation steps using simulated data 11

Web Figure 4. Number of infected individuals in the household by household size in A) in EpiCov households B) in simulated data, mean estimates after 1000 simulations, EpiCov study 2020 12

Web Table 1. Estimation of B and Q from synthetic datasets generated using the null model 13

Web Table 2. Size of the household in which individuals lived according to age 14

Web Table 3. Number of recruited and seropositive individuals by age and household size .15

Web Table 4. Comparison of model performance and estimated parameters: adjustment for characteristics of the susceptible individual..16

Web Table 5. Comparison of model performance and estimated parameters: adjustment for characteristics of the potential infector and family ties 17

Web Table 6. Comparison of model performance and estimated parameters: adjustment for socioeconomic characteristics 18

Web Table 7. Comparison of model performance and estimated parameters: adjustment for living conditions 19

Web Table 8. Comparison of model performance and estimated parameters: adjustment for region and immigration history 20

References 21

Web Appendix 1. The COVID-19 pandemic in France in 2020

The first wave of the COVID-19 pandemic peaked two weeks after the first national lockdown decreed from March 17 to May 11 2020 (Web Figure 1), in the context of mask shortages and little availability of PCR tests. This first lockdown combined drastic measures, including limited outdoor circulation, travel bans, mandatory teleworking, and the closure of schools, universities, and shops, except for essential supplies, which led to a very low incidence rate. The second wave started slowly at the end of August, despite wide-scale access to masks and free access to tests (both PCR and antigenic tests). Following a curfew period with territorial variations, a second national lockdown was reinstated from October 30 to December 15, 2020. This lockdown was less restrictive than the previous one, with no school closures (although universities were closed) and an extended list of shops authorized to remain open. Throughout the year, incentives for telework and other barrier measures, especially face covering and physical distancing, were maintained.

Web Appendix 2. Technical summary

We adopted an adapted version of chain-binomial models that fit the final size of infections (1), i.e., the distribution of seropositive and seronegative individuals within households, to analyze the transmission process among household members.

The model has been previously described by Bi et al. (2020)(2). We adapted their code available in open access to the EpiCov data.

The model estimates the risk of infection from: 1) extra-household sources and 2) a single infected household member.

Assumptions

The model's assumptions are as follows:

- each household member can be infected either from within a household or from extra-household sources
- household members mix at random within a household and can infect one another
- all household members were initially susceptible to infection to SARS-CoV-2
- the possibility of reinfection for the duration of the study period was neglected

In addition, we assumed no misclassification of the serological result, either positive or negative.

Data augmentation

Given that only the serological status of individuals was known, with no additional information about the chronology of infection events within the households, the model considers augmented data with all possible sequences of viral introductions to each household and subsequent transmission events within the household.

Possible sequences of viral introduction in the household and subsequent transmission events within the household are defined from the assignment of a generation to each household member. People infected from outside the household are assigned to generation 0. Those that they infect within the household are assigned to generation 1, those infected by generation 1 to generation 2, and so on. Uninfected individuals are assigned to generation infinity. For each household *h*, one *k* possible sequence $HH_{h,k}$ of viral introduction/transmission is one ordered assignment of generation.

For example, in a household of 3 individuals, *i, j,* and *k*, in which the 2 individuals *i* and *k* are positive and *j* is negative, there are three possible sequences (Web Figure 2).

In the first, HH_1, both *i* and *k* could have been infected outside of the household: the two are assigned to generation 0.

In the second, HH_2, *i* could have been infected outside and then infected k within the household: *i* is assigned to generation 0 and *k* to generation 1.

The third, HH_3, is the opposite: *k* could have been infected outside and then infected *i* within the household: *k* is assigned to generation 0 and *i* to generation 1.

In these three sequences, *j* is assigned to generation infinity.

It should be noted that several individuals can be assigned to the same generation i.e., one individual can infect several members of his/her household. In the previous example, if the three household members i, j and k are tested positive, we obtain the following sequences:

- {0,0,0}: the thee individuals are assumed to have become infected outside the household
- {0,1,1}, {1,1,0}, {1,0,1}: one individual, either i, j or k, is infected outside and then simultaneously infects the other two household members.
- {0,0,1}, {0,1,0}, {1,0,0}: two out of three individuals are infected outside and then one of them infects the third household member.
- {0,1,2}, {0,2,1}, {1,0,2}, {2,0,1}, {1,2,0}, {2,1,0}: one individual is infected outside the household, then infects one member of his household, who in turn infects the third individual.

Likelihood of the model

The likelihood of the model is calculated via the decomposition into the contribution of each possible sequence $HH_{h,k}$ of each household *h*.

The likelihood of the sequence $HH_{h,k}$ is: $\Pr(HH_{h,k}) = \prod_i \Pr(g_i|HH_{h,k})$

where $\Pr(g_i|\mathrm{HH}_{h,k})$ is the probability of household member *i* of household *h* having an infection generation of g_i in the sequence $\mathrm{HH}_{h,k}$

$\Pr(g_i|\mathrm{HH}_{h,k})$ is defined from the two probabilities of interest:

1) The probability $Q_{i,j}$ of a household member *i* escaping infection from a single infectious household member *j*, which corresponds to a person-to-person transmission probability.

2) The probability B_i of a household member *i* escaping infection from the community, i.e., extra-household exposure, over the course of epidemic.

$$\Pr(g_i|\mathrm{HH}_{h,k}) = (1 - B_i)^{I(g_i=0)}(B_i)^{I(g_i\neq 0)}[\prod_{j\neq i, g_j<(g_i-1)} Q_{i,j}][1 - \prod_{j\neq i, g_j=(g_i-1)} Q_{i,j}]$$

with $I := \begin{cases} 1, & g_i = 0 \\ 0, & g_i \neq 0 \end{cases}$

If i is infected outside, i.e., assigned to generation 0, it is simplified as $\Pr(g_i|\mathrm{HH}_{h,k}) = 1 - B_i$

If i is infected within the household, i.e., assigned to generation 1 or greater, it is simplified as

$$\Pr(g_i|\mathrm{HH}_{h,k}) = B_i[\prod_{j\neq i, g_j<(g_i-1)} Q_{i,j}][1 - \prod_{j\neq i, g_j=(g_i-1)} Q_{i,j}]$$

Where:

B_i represents the probability of household member *i* escaping infection from extra-household exposure

$\prod_{j\neq i, g_j<(g_i-1)} Q_{i,j}$ represents the probability of household member *i* escaping infection from other infected household members up to generation g_i

$1-\prod_{j\neq i, g_j=(g_i-1)} Q_{i,j}$ represents the probability of household member i being infected from any infected household members of generation $g_i - 1$

For each household h, the likelihood of observing the final infection state is the sum of the probability of all the possible sequences $HH_{h,k}$ that could lead to this final result.

$$\Pr(HH_h) = \sum_k \Pr(HH_{h,k})$$

The global log-likelihood of the model is the sum of the contribution of all households:

$$logLik = \sum_h \log\left(\Pr(HH_h)\right)$$

Covariates of adjustment

In a null model, the probabilities Q and B were fixed and equal for all individuals. Then, they were adjusted for individual and household characteristics.

$Q_{i,j}$ was estimated as a function of the exposed household member individual's characteristics $\boldsymbol{X_i}$, the potential infectors' characteristics $\boldsymbol{X_j}$, and some shared characteristics of their household $\boldsymbol{X_h}$ as follows:

$$logit(Q_{i,j}) = \beta_0 + X_i\beta + X_j\alpha + X_h\gamma$$

B_i, was estimated as a function of the exposed household member individual's characteristics $\boldsymbol{X_i}$ and his household's characteristics $\boldsymbol{X_h}$.

$$logit(B_i) = \beta_0' + X_i\beta' + X_h\gamma'$$

The probabilities of being infected from the community and from one single infected household member were then obtained as $1 - expit(logit(B_i))$ and $1 - expit(logit(Q_{i,j}))$ respectively, the expit function being the inverse of logit.

We consider the following covariates.

- **Covariates affecting susceptibility, $\boldsymbol{X_i}$**

Characteristics of the exposed (or susceptible) individual i
- age group (categorical): 6-10, 11-14, 15-17, 18-24, 25-34, 35-44, 45-54, 55-64, 65-74, ≥75
- gender (binary): female/male

- **Covariates affecting Infectivity, $\boldsymbol{X_j}$**

Characteristics of the potential infector j
- age group (categorical): 6-10, 11-14, 15-17, 18-24, 25-34, 35-44, 45-54, 55-64, 65-74, ≥75
- gender (binary): female/male

- family relationship with the individual *i* (categorical): partner/spouse, mother, father, child < 12 years old, child ≥ 12 years old, grandparent, grandchild < 12 years old, grandchild ≥ 12 years old, sibling < 12 years old, sibling ≥ 12 years old, other family link, no family link

- **Household-level covariates, $\boldsymbol{X_k}$**

- family income in deciles (categorical): D01 (lowest), D02-D03, D04-05, D06-07, D06-09, D10 (highest)
- density population in the municipality of residence (categorical): low, medium, high
- living in a socially deprived neighborhood (binary)
- accommodation type (categorical): an apartment with no balcony, terrace, or community garden, an apartment with a balcony or a terrace, an apartment with a community garden house with a yard or a garden, a house with a yard or a garden, other
- overcrowded housing, defined as less than 18 m^2 per inhabitant (binary)
- household size (categorical): 1, 2, 3, 4, ≥ 5 individuals
- region (categorical): the 13 administrative regions of mainland France
- immigration history (categorical): majority population, 1st-generation immigrant from Europe, 2nd-generation immigrant from Europe, 1st-generation immigrant from outside Europe, 2nd-generation immigrant from outside Europe. As the migration history was available only for the respondent member of the household and not all household members, this information was treated as a household-level covariate

Model selection

Associations of all the covariates mentioned with B_i and $Q_{i,j}$, respectively, were tested one by one in univariate models. In the final multivariate model, we adjusted for covariates for which a decrease was observed in the widely applicable information criterion (WAIC) and the leave-one-out cross-validation information criterion (LOOIC) in univariate analyses (3).

Inference and implementation

Posterior distributions of parameters were estimated via MCMC using the rstan package. The default algorithm in rstan is the No-U-Turn Sampler (NUTS), which is a tuning-free Hamiltonian-based Monte Carlo sampler (4).

We set weakly informative priors on all parameters to be normally distributed on the logit scale with a mean of 0 and a standard error of 1.5. We ran four chains of 1,500 iterations each, with 500 warm-up iterations, and assessed convergence visually and using the Gelman-Rubin Convergence Statistic (R-hat).

Handling of missing variables

Given the very low percentage of missing data for the considered variables (<4%), models were run using the complete dataset.

Validation

In order to evaluate the ability of our framework to estimate B and Q, we conducted a simulation study in which synthetic data generated on the basis of known values of B and Q were analyzed.

The different steps of the framework evaluation were as follows (Web Figure 3):

Step 1: Synthetic data generation:

We constructed a synthetic population of 500 households with the same household structure than our study population i.e., same proportions of households of various household size from 1 to 8.

For given fixed values of B and Q, we simulated the final distribution of cases, i.e. the number k of infected persons in each household of size n.

Simulation was performed according to the following algorithm, based on the null model on the null model where the same values of B and Q were applied to all individuals:

For each household with n susceptible individuals

1. Draw with probability (1-*B*) the individuals infected from extra-household exposures. They are assigned to generation 0 and $n\_inf\_g_0$ is the number of individuals assigned to generation 0.
2. If n = 1 OR $n\_inf\_g_0 = 0$. STOP
3. If n >1 AND $n\_inf\_g_0 > 0$, draw with probability ($1-Q^{n\_inf\_g0}$) the newly infected individuals within the household, infected by individuals of generation 0. These newly infected individuals are assigned to generation 1 and $n\_inf\_g_1$ is the number of individuals assigned to generation 1.
4. If $n\_inf\_g_i$, the number of individuals assigned to generation i, with i >1, is equal to 0. STOP Otherwise go to STEP3

   When the algorithm stops, the household members who remained uninfected are assigned to generation $g\infty$.

Step 2: Estimation of B and Q from the synthetic data

We ran our modeling framework to estimate B and Q from the synthetic data. Estimates and credible intervals (95% CrI) were then compared estimated values of B and Q with the known values set for generating the data.

These validation steps were applied for different sets of parameters B and Q. Results were very consistent, with all true values included in the estimated 95% CrI (Web Table 1).

Step 3: Adequacy of the simulation

We evaluated the adequacy of the simulated data with the final distribution of cases for the original data of the 8,165 households of the study population, for which serological status was available for all members and with no child aged ≤5 years.
We ran 1,000 simulations according to the algorithm presented above with the fixed parameters B = 0.955 and Q = 0.821, which were the B and Q values estimated from the main analysis.

In the simulated data, the overall seroprevalence was 6.8%, 95%CI (6.2 – 7.3), a slightly higher value than the reported seroprevalence observed in the EpiCov household data 6.2%, 95% CI (5.8 – 6.5).
The expected distribution of the number of infected individuals by household size is shown in Web Figure 4 and replicated the distribution of the EpiCov household data quite well.

Simulation of source of infection

For each household with at least one seropositive individual, we drew one sequence of viral introduction and subsequent within-household transmission from the probability distribution of all possible sequences of the household. We then estimated the number of infections acquired from extra-household exposure and the number of within-household transmission events in the drawn scenario.

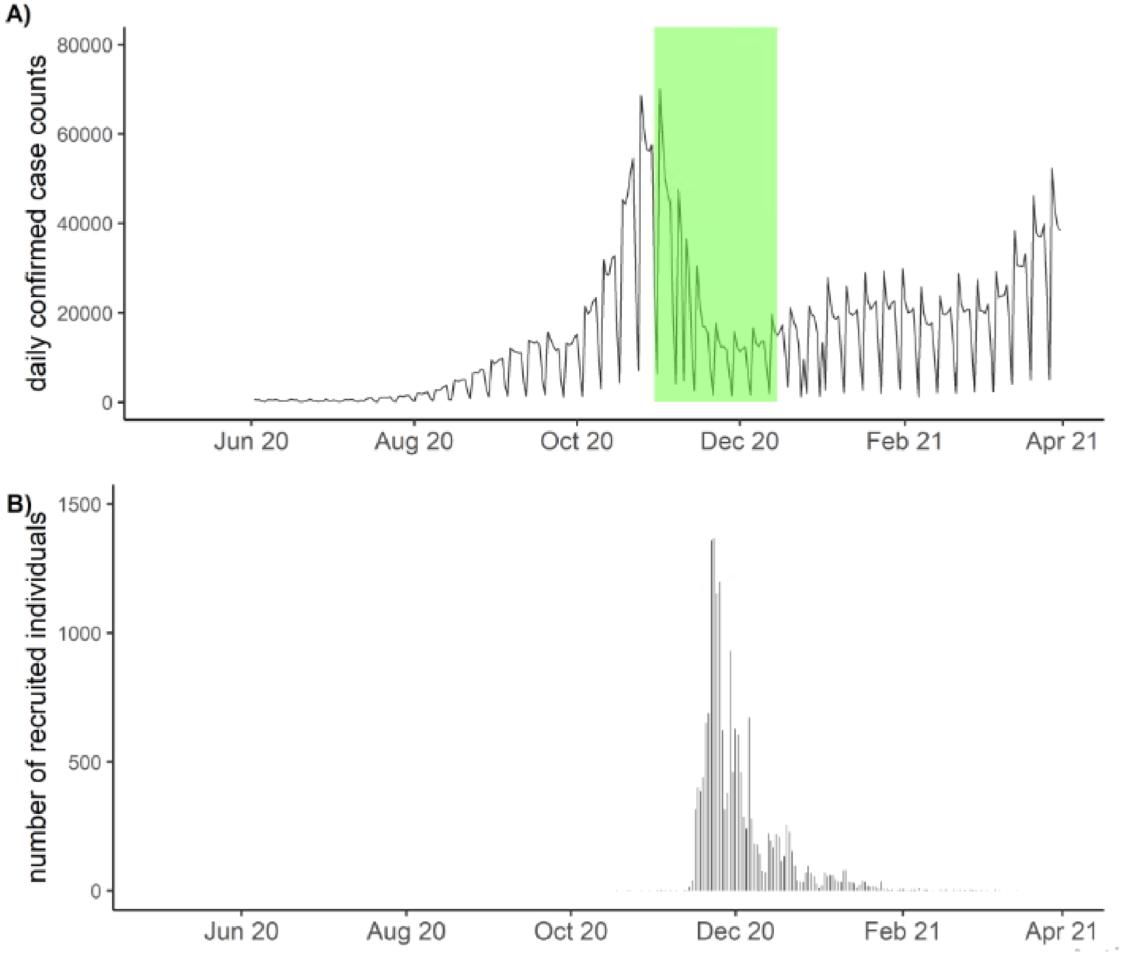

Web Figure 1. A) Number of reported cases in France in 2020 (source WHO Coronavirus (COVID-19) Dashboard (5) and B) recruitment period

The green area in the upper figure represents the lockdown period from October 31 to December 15 2020. It should be noted that population-based screening strategies and access to testing have increased significantly over the period presented, with very low screening before summer 2020.

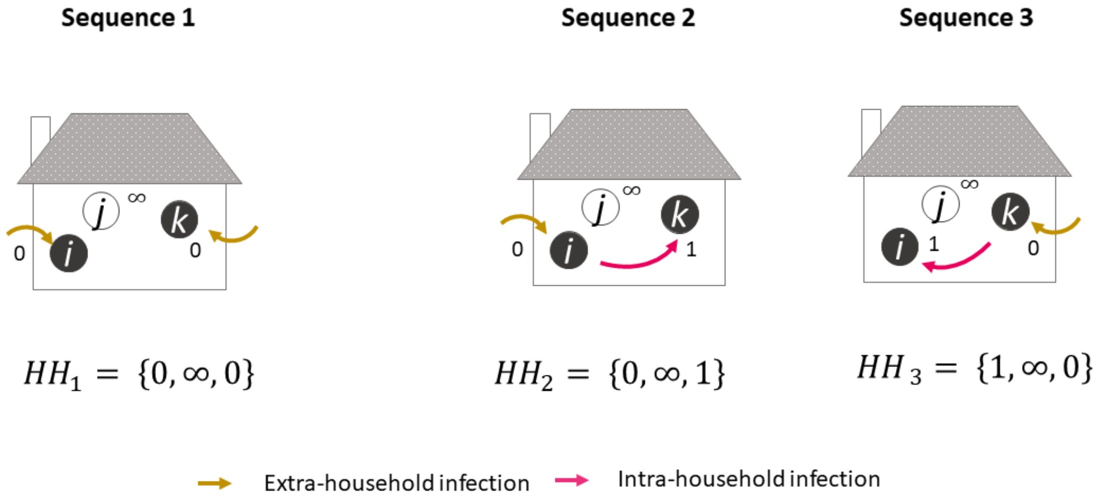

Web Figure 2. All possible sequences of viral introduction to each household and subsequent transmission events within the household for a household with three members with two positive individuals.

In a household of 3 individuals, *i, j,* and *k*, in which the 2 individuals *i* and *k* are positive and *j* is negative, there are three possible sequences.

In the first, HH_1, both *i* and *k* could have been infected outside of the household: the two are assigned to generation 0.

In the second, HH_2, *i* could have been infected outside and then infected *k* within the household: *i* is assigned to generation 0 and *k* to generation 1.

The third, HH_3, is the opposite: *k* could have been infected outside and then infected *i* within the household: *k* is assigned to generation 0 and *i* to generation 1.

In these three sequences, *j* is assigned to generation infinity.

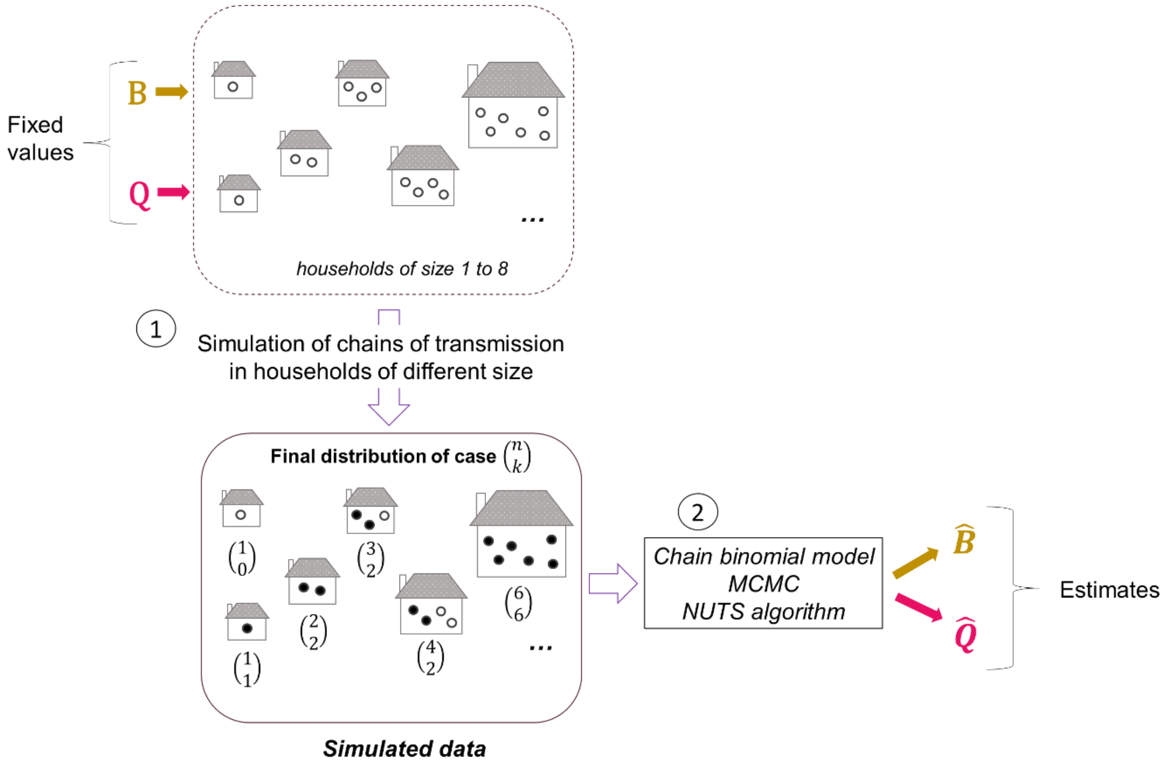

Web Figure 3. Model validation steps using simulated data

The framework evaluation consisted in two steps. 1) Synthetic data generation**:** we constructed a synthetic population of 500 households with the same household structure than our study population i.e., same proportions of households of various household size from 1 to 8. For given fixed values of B and Q, we simulated the final distribution of cases, i.e. the number k of infected persons in each household of size n. 2) Estimation of B and Q from the synthetic data: We ran our modeling framework to estimate B and Q from the synthetic data. Estimates and credible intervals (95% CrI) were then compared estimated values of B and Q with the known values set for generating the data.

These validation steps were applied for different sets of parameters B and Q.

Abbreviations: MCMC, Markov chain Monte Carlo; NUTS, No-U-Turn Sampler

A)

B)

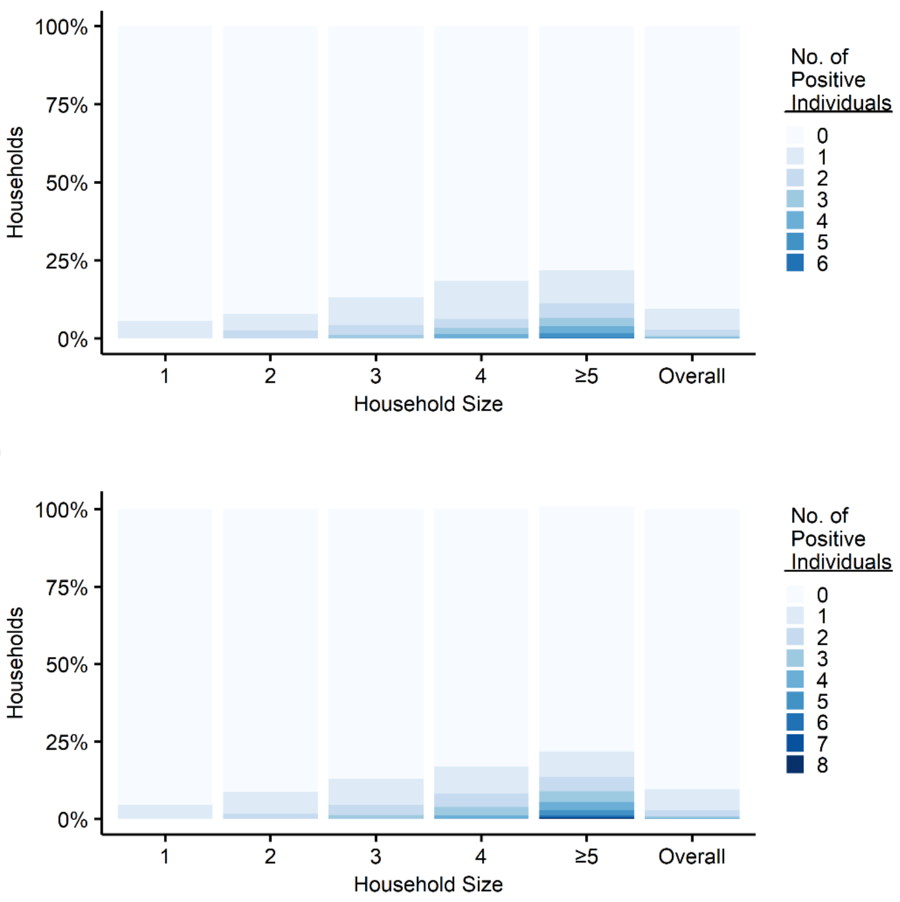

Web Figure 4. Number of infected individuals in the household by household size in A) in EpiCov households B) in simulated data, mean estimates after 1000 simulations, EpiCov study 2020

We simulated chains of transmission for the 8,165 households, for which serological status was available for all members and with no child aged ≤5 years, of the study population. Simulations were conducted 1,000 times with the fixed parameters B = 0.955 and Q = 0.821, which were the B and Q values estimated from the main analysis. We then combined the estimated mean number of infected individuals per household by household size.

Web Table 1. Estimation of B and Q from synthetic datasets generated using the null model

<u>Probabilities definitions:</u>

- B, probability that a household member escapes infection from extra-household exposure since the beginning of the pandemic to the time of the serosurvey
- Q, probability of a household member escaping infection from a single infectious household member, which corresponds to a person-to-person transmission probability.

Values of B and Q used to generate the simulated dataset	Estimated probabilities Median (95% Crl)
B = 0.90<br>Q = 0.90	B = 0.911 (0.891, 0.927)<br>Q = 0.927 (0.878, 0.970)
B = 0.80<br>Q = 0.20	B = 0.785 (0.756, 0.811)<br>Q = 0.202 (0.144, 0.268)
B = 0.20<br>Q = 0.80	B = 0.229 (0.191, 0.269)<br>Q = 0.788 (0.703, 0.876)
B = 0.50<br>Q = 0.50	B = 0.478 (0.437, 0.521)<br>Q = 0.469 (0.412, 0.531)
B = 0.20<br>Q = 0.20	B = 0.214 (0.172, 0.259)<br>Q = 0.212 (0.139, 0.295)

Abbreviations: 95% Crl, 95% credible interval

Web Table 2. Size of the household in which individuals lived according to age

	Age, years									
	**6-10**	**11-14**	**15-17**	**18-24**	**25-34**	**35-44**	**45-54**	**55-64**	**65-74**	**≥75**
	**N = 632**	**N = 832**	**N = 745**	**N = 1,797**	**N = 1,304**	**N = 1,513**	**N = 3,100**	**N = 3,601**	**N = 3,244**	**N = 1,215**
**Household size**										
**1**	0 (0)	0 (0)	0 (0)	10.0 (180)	21.1 (275)	13.2 (200)	8.8 (274)	13.1 (470)	14.2 (462)	21.0 (255)
**2**	3.2 (20)	2.9 (24)	4.6 (34)	14.5 (261)	51.5 (672)	21.5 (325)	23.7 (734)	62.0 (2,233)	79.2 (2,569)	71.4 (868)
**3**	17.1 (108)	14.9 (124)	18.7 (139)	24.2 (435)	14.2 (185)	15.9 (241)	24.1 (748)	16.6 (598)	5.4 (174)	5.8 (71)
**4**	53.3 (337)	54.3 (452)	50.1 (373)	34.2 (615)	9.4 (122)	37.7 (570)	32.3 (1,000)	6.6 (237)	0.9 (30)	1.0 (12)
**≥5**	26.4 (167)	27.9 (232)	26.7 (199)	17.0 (306)	3.8 (50)	11.7 (177)	11.1 (344)	1.7 (63)	0.3 (9)	0.7 (9)
**Family structure**										
**Living Alone**	0 (0)	0 (0)	0 (0)	10.0 (180)	21.1 (275)	13.2 (200)	8.8 (274)	13.1 (470)	14.2 (462)	21.0 (255)
**Couple Without Children**	0 (0)	0 (0)	0 (0)	7.2 (129)	46.9 (611)	17.8 (269)	19.4 (601)	58.9 (2,120)	76.8 (2,492)	68.0 (826)
**Single-Parent Family**	9.0 (57)	11.4 (95)	11.7 (87)	11.5 (206)	4.2 (55)	5.6 (84)	6.2 (192)	2.7 (99)	1.0 (3.1)	2.6 (32)
**Couple With One or more Children**	89.7 (567)	86.8 (722)	86.0 (641)	65.3 (1,173)	21.2 (277)	60.9 (922)	63.1 (1,956)	22.9 (826)	5.1 (164)	3.8 (46)
**3 Generation Family**	0.8 (5)	1.2 (10)	1.1 (8)	0.6 (10)	0.7 (9)	0.5 (8)	0.5 (17)	0.2 (9)	0.3 (9)	1.2 (14)
**Other Household Structure**	0.5 (3)	0.6 (5)	1.2 (9)	5.5 (99)	5.9 (77)	2.0 (30)	1.9 (60)	2.1 (77)	2.7 (86)	3.5 (42)

Results are shown as % (No.)

Web Table 3. Number of recruited and seropositive individuals by age and household size

Variable	Overall n / N (95% CI)	Size 1 n / N (95% CI)	Size 2 n / N (95% CI)	Size 3 n / N (95% CI)	Size 4 n / N (95% CI)	Size ≥5 n / N (95% CI)
**HOUSEHOLDS**	N = 8,165	N = 2,116	N = 3,870	N = 941	N = 937	N = 301
**≥ 1 seropositive**	784	120	302	124	172	66
	9.6 (9.0, 10.0)	5.7 (4.7, 6.8)	7.8 (7.0, 8.7)	13.0 (11.0, 16.0)	18.0 (16.0, 21.0)	22.0 (17.0, 27.0)
**> 1 seropositive**	229	0	95	41	59	34
	2.8 (2.5, 3.2)	-	2.5 (2.0, 3.0)	4.4 (3.2, 5.9)	6.3 (4.9,8.1)	11.0 (8.1, 16.0)
**INVIDIVUALS**	N = 17,983	N = 2,116	N = 7,740	N = 2,823	N = 3,748	N = 1,556
**Overall**	1,107	120	397	175	277	138
	6.2 (5.8, 6.5)	5.1 (4.7- 6.8)	5.1 (4.7, 5.7)	6.2 (5.4, 7.2)	7.4 (6.6, 8.3)	8.9 (7.5, 10.0)
**Age, years**						
**6-10**	35 / 632	0 / 0	1 / 20	5 / 108	19 / 337	10 / 167
	5.5 (3.9, 7.7)	-	5.0 (0.3, 27.0)	4.6 (1.7, 11.0)	5.6 (3.5, 8.8)	6.0 (3.1, 11.0)
**11-14**	62 / 832	0 / 0	2 / 24	12 / 124	27 / 452	21 / 232
	7.5 (5.8, 9.5)	-	8.3 (1.5, 28.0)	9.7 (5.3, 17.0)	6.0 (4.0, 8.7)	9.1 (5.8, 14.0)
**15-17**	57 / 745	0 / 0	5 / 34	7 / 139	26 / 373	19 / 199
	7.7 (5.9, 9.9)	-	15.0 (5.5, 32.0)	5.0 (2.2, 10.0)	7.0 (4.7, 10.0)	9.5 (6.0, 15.0)
**18-24**	192 / 1,797	21 / 180	22 / 261	45 / 435	66 / 615	38 / 306
	11.0 (9.3, 12.0)	12.0 (7.5, 17.0)	8.4 (5.5, 13.0)	10.0 (7.7, 14.0)	11.0 (8.5, 14.0)	12.0 (9.0, 17.0)
**25-34**	101 / 1,304	23 / 275	55 / 672	11 / 185	8 / 122	4 / 50
	7.7 (6.4, 9.4)	8.4 (5.5, 12.0)	8.2 (6.3, 11.0)	5.9 (3.2, 11.0)	6.6 (3.1, 13.0)	8.0 (2.6, 20.0)
**35-44**	95 / 1,513	9 / 200	19 / 325	11 / 241	45 / 570	11 / 177
	6.3 (5.1, 7.7)	4.5 (2.2, 8.6)	5.8 (3.7, 9.1)	4.6 (2.4, 8.2)	7.9 (5.9, 10.0)	6.2 (3.3, 11.0)
**45-54**	205 / 3,100	18 / 274	43 / 734	43 / 748	69 / 1,000	32 / 344
	6.6 (5.8, 7.6)	6.6 (4.1, 10.0)	5.9 (4.3, 7.9)	5.7 (4.2, 7.7)	6.9 (5.4, 8.7)	9.3 (6.5, 13.0)
**55-64**	180 / 3,601	22 / 470	111 / 2,233	30 / 598	14 / 237	3 / 63
	5.0 (4.3, 5.8)	4.7 (3.0, 7.1)	5.0 (4.1, 6.0)	5.0 (3.5, 7.2)	5.9 (3.4, 9.9)	4.8 (1.2, 14.0)
**65-74**	143 / 3,244	16 / 462	117 / 2,569	8 / 174	2 / 30	0 / 9
	4.4 (3.7, 5.2)	3.5 (2.1, 5.7)	4.6 (3.8, 5.5)	4.6 (2.2, 9.2)	6.7 (1.2, 24.0)	-
**≥75**	37 / 1,215	11 / 255	22 / 868	3 / 71	1 / 12	0 / 9
	3.0 (2.2, 4.2)	4.3 (2.3, 7.8)	2.5 (1.6, 3.9)	4.2 (1.1, 13.0)	8.3 (0.4, 40.0)	-

95% CI, 95% confidence interval [a] 16 missing values for gender

Web Table 4. Comparison of model performance and estimated parameters: adjustment for characteristics of the susceptible individual

Multiple models were run. They included key individual-level factors (i.e., age and sex of exposed individuals) that may be associated with risk of infection from extra-household exposures ('extra-household') and from a single infected household member ('intra-household'). Lower WAIC and LOOIC scores indicate better model fit.

	Model 1				Model 2		Model 3	
	Extra-household		Intra-household		Extra-household		Intra-household	
Variable	Age of the susceptible				Sex of the Susceptible		Sex of the Susceptible	
**Age of the Susceptible, years**	**OR**	**95%CrI**	**OR**	**95%CrI**	**OR**	**95%CrI**	**OR**	**95%CrI**
**6-10**	0.9	0.5, 1.5	0.4	0.2, 0.8				
**11-14**	1.2	0.8, 1.8	0.7	0.4, 1.3				
**15-17**	1.5	1.0, 2.2	0.7	0.3, 1.4				
**18-24**	2.5	2.0, 3.3	0.5	0.3, 0.9				
**25-34**	1.9	1.4, 2.5	0.5	0.2, 1.0				
**35-44**	1.3	1.0, 1.8	0.5	0.2, 0.9				
**45-54**	1.1	0.8, 1.4	0.8	0.5, 1.3				
**55-64**	1.0	Referent	1.0	Referent				
**65-74**	0.8	0.6, 1.1	2.7	1.5, 4.7				
**≥75**	0.7	0.5, 1.0	0.8	0.2, 2.0				
**Sex of the Susceptible**								
**Female**					1.0	Referent	1.0	Referent
**Male**					0.9	0.8, 1.1	0.7	0.5, 1.0
**N**	17,983				17,950		17,950	
**WAIC (p_waic)**	7,291.2 (19.8)				7,379.5 (3)		7,378.2 (2.9)	
**Delta WAIC / null model [a]**	-52.5				0.1		-0.6	
**LOOIC (p_loo)**	7,291.3 (19.9)				7,379.5 (3)		7,378.2 (2.9)	
**Delta LOOIC / null model [a]**	-52.4				0.1		-0.6	

Median 95% CrI: 95% Credible interval

Abbreviations: LOOIC, Leave-one-out information criterion; WAIC, Watanabe-Akaike information criterion; p_loo and p_waic, effective number of parameters for estimation of LOOIC and WAIC, respectively

[a] Difference in WAIC and LOOIC compared to the null model

Web Table 5. Comparison of model performance and estimated parameters: adjustment for characteristics of the potential infector and family ties

Multiple models were run. They included the age and sex of potential infectors, and family links between individuals that may be associated with risk of infection from a single infected household member ('intra-household'). Lower WAIC and LOOIC scores indicate better model fit.

	Model 4		Model 5		Model 6	
	Intra-Household		Intra-Household		Intra-Household	
**Variable**	Age of the Infector		Sex of the Infector		Family Ties	
**Age of the Infector, years**	**OR**	**95%CrI**	**OR**	**95%CrI**	**OR**	**95%CrI**
**6-10**	0.5	0.2, 1.1				
**11-14**	0.5	0.2, 1.1				
**15-17**	0.1	0.0, 0.3				
**18-24**	0.2	0.1, 0.3				
**25-34**	0.3	0.1, 0.7				
**35-44**	0.3	0.1, 0.6				
**45-54**	0.9	0.5, 1.4				
**55-64**	1.0	Referent				
**65-74**	1.5	0.8, 2.6				
**≥75**	0.7	0.2, 2.0				
**Sex of the Infector**						
**Female**			1.0	Referent		
**Male**			0.8	0.5, 1.3		
**Family Ties**						
**Between Partners**					1.0	Referent
**From Mother To Child**					1.0	0.6, 1.5
**From Father To Child**					0.4	0.1, 0.7
**From Child < 12 Years Old To Parent**					0.3	0.1, 0.8
**From Child ≥ 12 Years Old To Parent**					0.1	0.0, 0.2
**From Grandparent To Grandchild**					0.5	0.0, 4.8
**From Grandchild To Grandparent**					0.4	0.0, 2.6
**From A Sibling <12 Years Old**					0.4	0.1, 1.1
**From A Sibling ≥12 Years Old**					0.3	0.2, 0.6
**Between Individuals With Other Family Ties**					0.4	0.1, 1.3
**Between Individuals With No Family Ties**					0.1	0.0, 0.7
**N**	17,983		17,950		17,983	
**WAIC (p_waic)**	7,337.5 (10.8)		7,380.9 (3.4)		7,339.9 (8.8)	
**Delta WAIC / null model [a]**	-29.3		0.8		-28.1	
**LOOIC (p_loo)**	7,337.5 (10.8)		7,380.9 (3.4)		7,340 (8.9)	
**Delta LOO-PSIS / null model [a]**	-29.3		0.8		-28.1	

Median 95% CrI: 95% Credible interval

Abbreviations: LOOIC, Leave-one-out information criterion; WAIC, Watanabe-Akaike information criterion; p_loo and p_waic, effective number of parameters for estimation of LOOIC and WAIC, respectively

[a] Difference in WAIC and LOOIC compared to the null model

Web Table 6. Comparison of model performance and estimated parameters: adjustment for socioeconomic characteristics

Multiple models were run. They included household characteristics that may be associated with risk of infection from extra-household exposures ('extra-household') and from a single infected household member ('intra-household'). Lower WAIC and LOOIC scores indicate better model fit.

	Model 7		Model 8		Model 9		Model 10		Model 11	
	Extra-Household		Intra-Household		Extra-Household		Intra-Household		Extra-Household	
**Variable**	Family Income		Family Income		Population Density		Population Density		Socially Deprived Neighborhood	
**Family Income**	**OR**	**95% CrI**	**OR**	**95% CrI**	**OR**	**95% CrI**	**OR**	**95% CrI**	**OR**	**95% CrI**
D01 (lowest)	1.2	0.8, 1.8	0.5	0.2, 1.2						
D02-D03	1.0	0.7, 1.4	1.3	0.7, 2.3						
D04-D05	1.0	Referent	1.0	Referent						
D06-D07	1.2	0.9, 1.5	1.1	0.7, 1.9						
D08-D09	1.3	1.1, 1.7	1.1	0.7, 1.7						
D10 (highest)	1.7	1.3, 2.2	1.4	0.9, 2.3						
**Population Density in the Municipality of Residence**										
Low					1.0	Referent	1.0	Referent		
Medium					1.2	1.0, 1.4	1.0	0.7, 1.4		
High					1.6	1.4, 1.9	1.0	0.7, 1.4		
**Living in a Socially Deprived Neighborhood**										
No									1.0	Referent
Yes									1.0	0.6, 1.7
**N**	17,704		17,704		17,983		17,983		17,983	
**WAIC (p_waic)**	7,261.7 (7)		7,277.2 (7.4)		7,366.3 (4.1)		7,400.4 (4.4)		7,398 (3.1)	
**Delta WAIC / null model [a]**	-6.3		1.5		-14.9		2.1		0.9	
**LOOIC (p_loo)**	7,261.7 (7)		7,277.2 (7.4)		7,366.3 (4.1)		7,400.4 (4.4)		7,398 (3.1)	
**Delta LOO-PSIS / null model [a]**	-6.3		1.5		-14.9		2.1		0.9	

Median 95% CrI: 95% Credible interval

Abbreviations: LOOIC, Leave-one-out information criterion; WAIC, Watanabe-Akaike information criterion; p_loo and p_waic, effective number of parameters for estimation of LOOIC and WAIC, respectively.

[a] Difference in WAIC and LOOIC compared to the null model

Web Table 7. Comparison of model performance and estimated parameters: adjustment for living conditions

Multiple models were run. They included household characteristics that may be associated with risk of infection from extra-household exposures ('extra-household') and from a single infected household member ('intra-household'). Lower WAIC and LOOIC scores indicate better model fit.

	Model 11		Model 12		Model 13	
	Intra-Household		Intra-Household		Intra-Household	
**Variable**	**Accommodation Type**		**Household Size**		**Overcrowded Housing**	
**Accommodation type**	**OR**	**95% CrI**	**OR**	**95% CrI**	**OR**	**95% CrI**
**A House with a Yard or a Garden**	1.0	Referent				
**A House with No Yard or Garden**	1.5	0.4, 4.0				
**An Apartment with a Balcony or a Terrace**	1.3	0.9, 1.8				
**An Apartment with a Community Garden**	0.8	0.1, 2.5				
**An Apartment with No Balcony, Terrace or Community Garden**	1.1	0.5, 2.1				
**Other**	0.9	0.2, 3.1				
**Household size**						
**2**			1.0	Referent		
**3**			0.5	0.3, 0.7		
**4**			0.4	0.3, 0.6		
**≥5**			0.4	0.3, 0.6		
**Overcrowded housing [a]**						
**Crowded Housing**					1.2	0.8, 1.7
**Housing Not Particularly Crowded**					1.0	Referent
**N**	17,980		17,983		16,883	
**WAIC (p_waic)**	7,403 (7)		7,373.3 (5.4)		6,739.2 (3.3)	
**Delta WAIC / null model [b]**	3.6		-11.4		0.7	
**LOOIC (p_loo)**	7,403.3 (7.1)		7,373.3 (5.5)		6,739.2 (3.3)	
**Delta LOO-PSIS / null model [b]**	3.7		-11.4		0.7	

Median 95% CrI: 95% Credible interval

Abbreviations: LOOIC, Leave-one-out information criterion; WAIC, Watanabe-Akaike information criterion; p_loo and p_waic, effective number of parameters for estimation of LOOIC and WAIC, respectively

[a] Overcrowded housing defined as at least two people living in less than 18 m2 per person

[b] Difference in WAIC and LOOIC compared to the null model

Web Table 8. Comparison of model performance and estimated parameters: adjustment for region and immigration history

Multiple models were run. They included household characteristics that may be associated with risk of infection from extra-household exposures ('extra-household') and from a single infected household member ('intra-household'). Lower WAIC and LOOIC scores indicate better model fit.

	Model 15		Model 16		Model 17	
	Extra-Household		Extra-Household		Intra-Household	
**Variable**	**Region**		**Immigration History**		**Immigration History**	
**Region**	**OR**	**95% Crl**	**OR**	**95% Crl**	**OR**	**95% Crl**
**Auvergne-Rhone-Alpes**	1.9	1.3, 2.9				
**Bourgogne-Franche-Comte**	1.1	0.7, 1.9				
**Bretagne**	0.6	0.3, 1.0				
**Centre-Val de Loire**	1.0	Referent				
**Corse**	0.2	0.0, 1.2				
**Grand Est**	1.5	1.0, 2.3				
**Hauts-de-France**	1.8	1.2, 2.7				
**Ile-de-France**	2.3	1.6, 3.5				
**Normandie**	1.1	0.7, 1.9				
**Nouvelle-Aquitaine**	0.8	0.5, 1.3				
**Occitanie**	0.9	0.6, 1.4				
**Pays de la Loire**	1.0	0.6, 1.6				
**Provence-Alpes-Côte d'Azur**	0.8	0.5, 1.4				
**Immigration history of the respondent**						
**Majority population**			1.0	Referent	1.0	Referent
**1st Generation Immigrant from Europe**			0.8	0.5, 1.2	1.0	0.4, 2.1
**1st Generation Immigrant from Outside Europe**			1.6	1.0, 2.4	0.6	0.2, 1.2
**2nd Generation Immigrant from Europe**			1.0	0.7, 1.4	1.3	0.7, 2.3
**2nd Generation Immigrant from Outside Europe**			1.6	1.0, 2.3	1.3	0.7, 2.4
**N**	17,983		17,442		17,442	
**WAIC (p_waic)**	7,287.2 (12.9)		7,187.2 (6.1)		7,196 (7.6)	
**Delta WAIC / null model [a]**	-54.5		-1.3		3.0	
**LOOIC (p_loo)**	7,287.2 (12.9)		7,187.2 (6.1)		7,196.2 (7.7)	
**Delta LOO-PSIS / null model [a]**	-54.5		-1.3		3.1	

Median 95% Crl: 95% Credible interval

Abbreviations: LOOIC, Leave-one-out information criterion; WAIC, Watanabe-Akaike information criterion; p_loo and p_waic, effective number of parameters for estimation of LOOIC and WAIC, respectively

[a] Difference in WAIC and LOOIC compared to the null model

References

1. Longini IM, Koopman JS. Household and Community Transmission Parameters from Final Distributions of Infections in Households. *Biometrics*. 1982;38(1):115.

2. Bi Q, Lessler J, Eckerle I, et al. Insights into household transmission of SARS-CoV-2 from a population-based serological survey. *Nat Commun*. 2021;12(1):3643.

3. Watanabe S. Asymptotic Equivalence of Bayes Cross Validation and Widely Applicable Information Criterion in Singular Learning Theory. *Journal of Machine Learning Research*. 2010;11(116):3571–3594.

4. Hoffman MD, Gelman A. The No-U-Turn Sampler: Adaptively Setting Path Lengths in Hamiltonian Monte Carlo. *Journal of Machine Learning Research*. 2014;15(47):1593–1623.

5. WHO Coronavirus (COVID-19) Dashboard | WHO Coronavirus (COVID-19) Dashboard
